# Clinical and Economic Outcomes in Low-risk Pulmonary Embolism Patients Treated with Rivaroxaban versus Standard of Care

**DOI:** 10.36469/9936

**Published:** 2019-10-02

**Authors:** W. Frank Peacock, Craig I. Coleman, Phil Wells, Gregory J. Fermann, Li Wang, Onur Baser, Jeff Schein, Concetta Crivera

**Affiliations:** 1 Baylor College of Medicine; 2 University of Connecticut; 3 University of Ottawa and the Ottawa Hospital Research Institute; 4 University of Cincinnati; 5 STATinMED Research; 6 The University of Michigan; 7 Janssen Scientific Affairs, LLC

**Keywords:** standard of care, rivaroxaban, pulmonary embolism, outcomes research

## Abstract

**Background:**

Rivaroxaban, a fixed-dose oral direct factor Xa inhibitor, does not require continuous monitoring and thus reduces the hospital stay and economic burden in low-risk pulmonary embolism (LRPE) patients.

**Study Question:**

What is the effectiveness of rivaroxaban versus the standard of care (SOC; low-molecular-weight heparin, unfractionated heparin, warfarin) among LRPE patients in the Veterans Health Administration?

**Study Design:**

Adult patients with continuous health plan enrollment for ≥12 months pre- and 3 months post-inpatient PE diagnosis (index date=discharge date) between October 1, 2011- June 30, 2015 and an anticoagulant claim during the index hospitalization were included.

**Measures and Outcomes:**

Patients scoring 0 points on the simplified Pulmonary Embolism Stratification Index were considered low-risk and were stratified into SOC and rivaroxaban cohorts. Propensity score matching (PSM) was used to compare hospital-acquired complications (HACs), PE-related outcomes (recurrent venous thromboembolism, major bleeding, and death), and healthcare utilization and costs between the rivaroxaban and SOC cohorts.

**Results:**

Among 6746 PE patients, 1918 were low-risk; of these, 73 were prescribed rivaroxaban, 1546 were prescribed SOC, and 299 were prescribed other anticoagulants during the index hospitalization. After 1:3 PSM, 64 rivaroxaban and 192 SOC patients were included. During the index hospitalization, rivaroxaban users (versus SOC) had similar inpatient length of stay (LOS; 7.0 vs 6.7 days, standardized difference [STD]=1.8) but fewer HACs (4.7% vs 10.4%; STD: 21.7). In the 90-day post-discharge period, PE-related outcome rates were similar between the cohorts (all p>0.05). However, rivaroxaban users had fewer outpatient (15.9 vs 20.4; p=0.0002) visits per patient as well as lower inpatient ($765 vs $2,655; p<0.0001), pharmacy ($711 vs $1,086; p=0.0033), and total costs ($6,270 vs $9,671; p=0.0027).

**Conclusions:**

LRPE patients prescribed rivaroxaban had similar index LOS and PE-related outcomes, but fewer HACs, and lower total costs than those prescribed SOC.

## Introduction

Pulmonary embolism (PE) is a common form of venous thromboembolism (VTE) and is defined as a mechanical obstruction in the pulmonary artery or its branches with a blood clot, tumor, air, or fat.^1,2^ Among patients with vascular disease, PE is the third most common cardiovascular event behind myocardial infarction and stroke, with an annual rate of 112 cases per 100 000 that rises with age.^2,3^ The mortality rate in PE patients is estimated to be 10% at 1-3 months, with the highest mortality occurring in those presenting with hypotension and evidence of right ventricular dysfunction.^4–6^ In the United States, PE causes 100 000 deaths annually; in Europe, PE-related deaths were estimated at 300 000 deaths annually. The economic burden of PE is also substantial. In 2015, the estimated mean daily per patient hospitalization costs were $1735, and the total annual initial PE hospitalization cost was $11 486 per patient in the United States.^7^

A majority of PE patients are treated with a vitamin K antagonist (VKA; ie, warfarin) bridged with a parenteral anticoagulant of either low-molecular-weight heparin (LMWH) or unfractionated heparin (UFH) as the standard of care (SOC) therapy.^8–10^ Although the effectiveness of warfarin is well-established, the drug requires more time to achieve optimal therapeutic anticoagulation (≥5 days).^11^ Warfarin use also has several limitations, including frequent laboratory monitoring, dose adjustments, and numerous medication and dietary interactions.^8^ Rivaroxaban is a fixed-dose oral direct factor Xa inhibitor approved by the Food and Drug Administration in 2011 with fewer food and drug interactions compared with SOC drugs.^10^ Additionally, it has a quicker onset of action—about 2-4 hours after initiation—and no requirement for coagulation monitoring.^11,12^ By eliminating the temporal necessity for bridging, rivaroxaban can potentially reduce the hospital length of stay (LOS), thus reducing economic burden in PE patients.^13–16^ Additionally, MERCURY PE and HOT PE trials have evaluated the efficiency of rivaroxaban in the outpatient management of low-risk PE patients (LRPE).^17,18^

The European Society of Cardiology advocates for the risk stratification of patients with PE and the consideration of outpatient management options for patients with LRPE. Additionally, patients with LRPE may qualify for immediate or early discharge.^16^

However, US physicians have not widely adopted an outpatient or observation management strategy.^19–21^ Some factors identified as barriers to outpatient management of LRPE patients include physician resistance, medication security, difficulty in risk stratification, and a lack of uniform approach to risk stratification.^19^ Several risk-stratification algorithms have been developed; the Pulmonary Embolism Severity Index (PESI) and the simplified PESI (sPESI) scores are extensively validated prognostic tools.^22^ However, there is a paucity of research on the impact of rivaroxaban among LRPE patients using real-world data. Therefore, our purpose was to evaluate the effectiveness of rivaroxaban versus SOC among LRPE patients in the Veterans Health Administration (VHA) population.

## Material and Methods

### Data Source

This was a longitudinal, retrospective cohort study assessing the VHA population from October 1, 2010 to September 30, 2015. The VHA is the largest integrated healthcare system in the United States, providing care at 1245 healthcare facilities, including 170 VA medical centers and 1065 outpatient clinics, serving more than 9 million enrolled veterans across the country.^23^

Electronic health data collected within the VHA national Medical Statistical Analysis System (SAS) Dataset and Decision Support System were evaluated using the medical, pharmacy, laboratory, and VHA health plan enrollment information.^24,25^ These data include hospital and outpatient diagnoses (International Classification of Diseases, 9th Revision, Clinical Modification [ICD-9-CM]) and procedure codes (ICD-9 procedure and Current Procedural Terminology codes)^26^, laboratory results, and dispensed medication records. Death date was determined using the VA Vital Status file, which ascertains mortality using the Social Security Death Master File, Medicare Vital Status Files, and VA Beneficiary Identification and Records Locator Subsystem. The VHA Vital Status File is updated quarterly, and the three most recent quarterly updates are maintained.^27^

### Study Population

**Figure 1.**
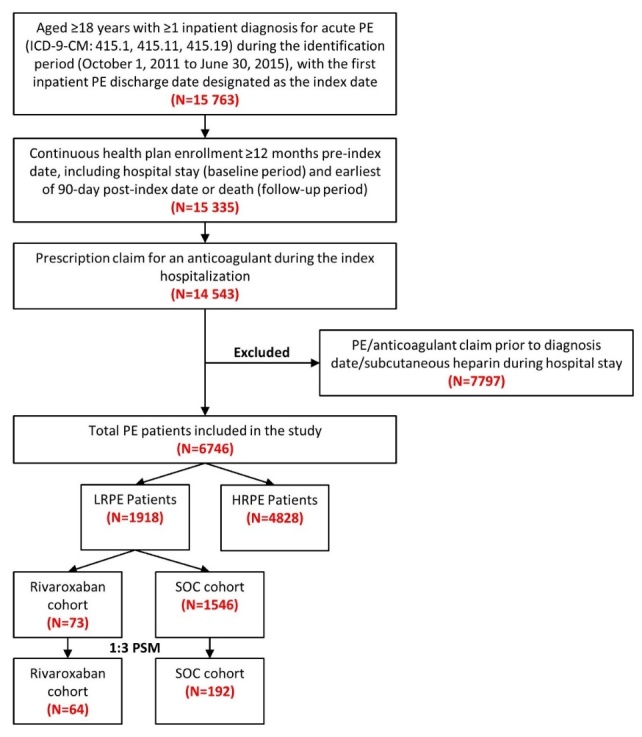
Study Design International Classification of Diseases, 9th Revision, Clinical Modification; PE: pulmonary embolism; PSM: propensity score matching; SOC: standard of care

Patients were included in the study if they had ≥1 inpatient diagnosis claim for PE (ICD-9-CM codes 415.1, 415.11, 415.19) during the identification period (October 1, 2011 to June 30, 2015), with the first admission date for a PE claim considered as the initial diagnosis date and the hospital discharge date designated as the index date (Figure 1). Included patients were ≥18 years of age, had an anticoagulant claim (UFH, LMWH, warfarin, or novel oral anticoagulant [NOAC]) during their index hospitalization, and were continuously enrolled in their health plan with medical and pharmacy benefits for ≥12 months prior to the index hospitalization discharge date, including the hospital stay (baseline period) until 3 months post-index date or death (follow-up period), whichever occurred first. Patients administered subcutaneous heparin during their hospital stay were not included, since many patients are given subcutaneous heparin as a prophylaxis for deep vein thrombosis and PE. Patients with a PE claim or any anticoagulant claim (UFH, LMWH, warfarin, or NOACs) prior to the initial diagnosis date were excluded.

Eligible PE patients were stratified using the sPESI criteria into LRPE and high-risk PE (HRPE) cohorts. The sPESI is a simplified version of the PESI, in which selected variables of the original score are included (age, history of cancer, history of chronic cardiopulmonary disease, pulse, systolic blood pressure, and oxygen saturation). Patients with a score of 0 were considered low-risk; all others were considered high-risk. LRPE patients were further stratified into rivaroxaban and SOC cohorts based on the presence of a prescription claim for an anticoagulant on the index date. SOC drugs included LMWH or UFH and warfarin. Patients in the SOC cohort did not have a rivaroxaban claim during the index hospitalization.

### Ethics Approval

Since the core study herein did not involve the collection, use, or transmittal of individual identifiable data, Institutional Review Board approval to conduct this study was not required.

### Baseline Measures

Patient demographics including age, sex, race and body mass index during the baseline period were assessed. In addition, clinical characteristics including various diagnostic tests, Charlson comorbidity index (CCI) scores, individual comorbidities (hospitalized deep vein thrombosis [DVT; ICD-9-CM codes 451.1, 453], left ventricular [LV] dysfunction [ICD-9-CM code 429.9], and cardiac dysrhythmia [ICD-9-CM codes 427.0-427.9]) were recorded. Further, the percentage of patients with hospital-acquired complications (HACs) and various clinical marker testing (troponin I/T, B type natriuretic peptide [BNP], and NT-pro BNP) during the index hospitalization was evaluated. HACs included any of the following conditions using pre-specified ICD-9-CM codes (available upon request): catheter-associated urinary tract infection, methicillin-resistant *Staphylococcus aureus*, *Clostridium difficile* infection, hospital-acquired bacterial pneumonia, foreign object retained after surgery, air embolism, blood incompatibility, pressure ulcer (stages III & IV), trauma/injury, procedure-related complications, poor glycemic control, iatrogenic pneumothorax with venous catheterization, vascular catheter-associated infection, or surgical site infection.

### Outcome Measures

PE-related clinical outcomes (recurrent VTE, major bleeding, or death), and diagnostic tests including computed tomography angiography (CTA), Echocardiogram (ECHO), lung ventilation/perfusion (VQ) scan, and Venous Doppler Ultrasound during the 90-day post-discharge period were evaluated. The percentage of patients with any (i.e., not disease-specific) inpatient hospitalizations and outpatient stays were reported. The mean number of visits per patient and associated healthcare costs (inpatient, outpatient and pharmacy) during the 90-day follow-up period were also reported. Direct medical and pharmacy costs were only evaluated in our study using the corresponding costs directly available in the VA data. Also, these medical and pharmacy costs were only from the VA perspective. Costs were adjusted to 2015 US dollars, using the medical care component of consumer price index (CPI) to reflect inflation.

### Statistical Analysis

Descriptive statistics were provided for all study variables—including baseline demographics, clinical characteristics, and outcome variables—among rivaroxaban and SOC cohorts. Statistical tests of significance (chi-square for categorical variables, t-test for continuous variables) were conducted to assess differences between the cohorts. Propensity score matching (PSM) was used to compare clinical and economic outcomes among the cohorts. Each rivaroxaban patient was matched to three SOC patients within 0.01 units of the propensity score. The propensity score was calculated via a logistic regression model. Variables adjusted in the PSM model included sex, race, clinical characteristics/markers, baseline diagnostic tests, sPESI score, cardiac dysrhythmia, LV dysfunction, hospitalized DVT, and CCI-related individual comorbidities. The matching procedure’s adequacy was assessed using standardized difference (STD); a difference of <10% was considered well-balanced.^28^ Healthcare resource utilization (HRU) and costs were compared between the PSM-matched cohorts and the p-values were calculated from a generalized linear model (GLM). All analyses were conducted using SAS statistical software (Version 9.3, SAS Institute, Cary, North Carolina, 2012).

## Results

After applying the inclusion and exclusion criteria, 6746 PE patients were included in the study, among which 1918 (28.4%) were stratified as LRPE patients. Among the LRPE patients, 73 (3.8%) received rivaroxaban, 1546 (80.6%) received SOC, and 299 (15.6%) received other anticoagulants during index hospitalization (Figure 1).

Note: The sum of the patients in the rivaroxaban and SOC cohorts is not equal to the total number of LRPE patients (1,918) because patients were prescribed other anticoagulants during the inpatient stay.

### Patient Characteristics during the Baseline Period:

#### Before Matching

The average age of patients in both cohorts was approximately 60 years, and the majority were male (97.3% vs 93.1%, STD: 19.6) and white (57.5% vs 64.7%, STD:14.6). The rivaroxaban cohort had a lower mean CCI score (0.6 vs 1.0; STD: 35.1) and a lower proportion of patients with diabetes (16.4% vs 26.8%; STD=25.3). Additionally, the rivaroxaban cohort had a shorter inpatient length of stay (LOS; 6.2 vs 8.2, STD:12.4), lower proportion of patients with HACs (5.5% vs. 10.0%, STD: 17.0) and higher proportion of patients with BNP measured during their index hospitalization (38.4% vs 25.3%, STD: 28.2; Table 1).

**Table 1.** Demographic and Clinical Characteristics of LRPE Patients prescribed SOC and Rivaroxaban -Baseline and Index hospitalization

**Baseline Demographic and Clinical Characteristics**	**Before PSM Matching**					**After 1:3 PSM Matching**				
	**SOC Cohort**		**Rivaroxaban Cohort**			**SOC Cohort**		**Rivaroxaban Cohort**		
	**N=(1546)**		**N=(73)**			**N=(192)**		**N=(64)**		
	**N/Mean**	**%/STD**	**N/Mean**	**% SD**	**STD**	**N/Mean**	**%/SD**	**N/Mean**	**% SD**	**STD**
**Age**										
**Mean, SD**	59.9	11.1	59.8	11.7	0.3	57.8	11.2	60.2	11.9	20.4
**Median**	62		63			59.5			63	
18-45	169	10.9%	9	12.3%	4.3	27	14.1%	9	14.1%	0.0
46-64	788	51.0%	31	42.5%	17.0	106	55.2%	25	39.1%	32.6
65+	589	38.1%	33	45.2%	14.4	59	30.7%	30	46.9%	33.4
**Gender**										
Male	1439	93.1%	71	97.3%	19.6	185	96.4%	62	96.9%	2.9
**Race**										
White	1000	64.7%	42	57.5%	14.6	129	67.2%	40	62.5%	9.8
Black	400	25.9%	22	30.1%	9.5	49	25.5%	17	26.6%	2.4
Unknown	101	6.5%	7	9.6%	11.2	8	4.2%	5	7.8%	15.3
Other	45	2.9%	2	2.7%	1.0	6	3.1%	2	3.1%	0.0
**Body Mass Index**										
Body Mass Index (in kg/m**^2^)**	31.7	9.5	32.3	6.4	7.5	32.2	7.3	32.6	6.7	5.2
**Baseline Comorbid Conditions**										
**Charlson Comorbidity Index Score**	1.0	1.4	0.5	0.9	35.1	0.4	0.8	0.5	0.9	12.2
Myocardial Infarction	72	4.7%	3	4.1%	2.7	3	1.6%	2	3.1%	10.3
Congestive heart failure	0	0.0%	0	0.0%	0.0	0	0.0%	0	0.0%	0.0
Peripheral vascular disease	84	5.4%	3	4.1%	6.2	9	4.7%	3	4.7%	0.0
Dementia	11	0.7%	0	0.0%	12.0	0	0.0%	0	0.0%	0.0
Cerebrovascular disease	133	8.6%	3	4.1%	18.4	4	2.1%	3	4.7%	14.3
Chronic pulmonary disease	108	7.0%	6	8.2%	4.6	17	8.9%	5	7.8%	3.8
Rheumatologic disease or connective tissue disease	23	1.5%	0	0.0%	17.4	0	0.0%	0	0.0%	0.0
Peptic Ulcer disease	26	1.7%	1	1.4%	2.5	2	1.0%	1	1.6%	4.6
Mild liver disease	15	1.0%	0	0.0%	14.0	0	0.0%	0	0.0%	0.0
Hemiplegia or paraplegia	0	0.0%	0	0.0%	0.0	0	0.0%	0	0.0%	0.0
Moderate or severe renal disease	270	17.5%	8	11.0%	12.6	8	4.2%	6	9.4%	14.3
Diabetes	414	26.8%	12	16.4%	25.2	30	15.6%	11	17.2%	4.2
Any tumor	12	0.8%	0	0.0%	8.8	2	1.0%	0	0.0%	10.2
Moderate or severe liver disease	12	0.8%	0	0.0%	7.2	0	0.0%	0	0.0%	0.0
Metastatic solid tumor	0	0.0%	0	0.0%	0.0	0	0.0%	0	0.0%	0.0
Diabetes + complications	182	11.8%	4	5.5%	15.5	10	5.2%	4	6.3%	3.1
AIDS	120	7.8%	0	0.0%	16.2	0	0.0%	0	0.0%	0.0
Cardiac Dysrhythmia	224	14.5%	6	8.2%	19.8	18	9.4%	6	9.4%	0.0
LV dysfunction	34	2.2%	1	1.4%	6.2	0	0.0%	1	1.6%	17.7
Hospitalized DVT	525	34.0%	24	32.9%	2.3	80	41.7%	21	32.8%	18.3
**Baseline Diagnostic tests**										
CTA	809	52.3%	43	58.9%	13.2	113	58.9%	37	57.8%	2.1
ECHO	32	2.1%	2	2.7%	4.4	4	2.1%	0	0.0%	20.6
VQ Scan	37	2.4%	0	0.0%	22.1	0	0.0%	0	0.0%	0.0
Venous Doppler Ultrasound	314	20.3%	12	16.4%	10.0	43	22.4%	9	14.1%	21.6
**Clinical Characteristics During Index Hospitalization**										
**Index hospital Length of stay (days)**	8.2	15.6	6.2	16.3	12.4	7.0	19.8	6.7	21.8	1.8
**Hospital acquired complications (HACs) during index hospitalization, ANY**	155	10.0%	4	5.5%	17.0	20	10.4%	3	4.7%	21.7
Catheter-associated Urinary Tract Infection	4	0.3%	0	0.0%	7.2	0	0.0%	0	0.0%	0.0
Methicillin-resistant Staphylococcus Aureus (MRSA)	6	0.4%	0	0.0%	8.8	0	0.0%	0	0.0%	0.0
Clostridium Difficile Infection	9	0.6%	0	0.0%	10.8	0	0.0%	0	0.0%	0.0
Hospital Acquired (Bacterial) Pneumonia	94	6.1%	3	4.1%	8.9	9	4.7%	2	3.1%	8.0
Foreign Object Retained After Surgery	1	0.1%	0	0.0%	3.6	0	0.0%	0	0.0%	0.0
Pressure Ulcer Stages III & IV	2	0.1%	0	0.0%	5.1	0	0.0%	0	0.0%	0.0
Trauma/Injury	45	2.9%	1	1.4%	10.6	10	5.2%	1	1.6%	20.2
Poor Glycemic Control	7	0.5%	0	0.0%	9.5	1	0.5%	0	0.0%	10.2
Vascular Catheter-associated Infection	1	0.1%	0	0.0%	3.6	0	0.0%	0	0.0%	0.0
Surgical Site Infection	2	0.1%	0	0.0%	5.1	0	0.0%	0	0.0%	0.0
Bacterial Pneumonia	154	10.0%	3	4.1%	23.0	14	7.3%	2	3.1%	18.8
**Clinical marker testing during the index hospitalization**										
# Patients with Troponin I	558	36.1%	20	27.4%	18.7	52	27.1%	17	26.6%	1.2
# Patients with Troponin T	29	1.9%	1	1.4%	4.0	3	1.6%	1	1.6%	0.0
# Patients with BNP	391	25.3%	28	38.4%	28.2	69	35.9%	22	34.4%	3.3
# of Patients with NT Pro BNP	150	9.7%	9	12.3%	8.4	17	8.9%	7	10.9%	6.9

BNP: B type natriuretic peptide; CTA: computed tomography angiography; DVT: deep vein thrombosis; ECHO: echocardiogram; LV: left ventricular; LRPE: low-risk pulmonary embolism; SD: standard deviation; SOC: standard of care; STD: standardized difference; VQ: lung ventilation/perfusion.

#### After PSM Matching

After 1:3 PSM, 64 LRPE patients were included in the rivaroxaban cohort and 192 LRPE patients were included in the SOC cohort. (Table 1). During index hospitalization, the rivaroxaban cohort had similar index LOS (7.0 vs 6.7 days, STD: 1.8) but a lower proportion of patients with HACs (4.7% vs 10.4%; STD: 21.7) as compared to SOC cohort (Table 1).

### PSM-adjusted Outcomes during the 90-day Follow-up Period

There were no statistically significant differences in follow-up PE-related clinical outcomes between the rivaroxaban and SOC cohorts, including recurrent VTE (3.1% vs 4.7%, p=0.5935), major bleeding (0.0% vs 1.0%, p=0.4124), and death (0.0% vs 1.6%, p=0.3145), respectively. No differences between the cohorts were found in the proportion of patients with various diagnostic tests (including CTA, ECHO, VQ scan, venous Doppler ultrasound) during the 90-day follow-up period (Table 2).

**Table 2.** PSM-adjusted Outcomes among LRPE Patients Prescribed SOC Therapy versus Rivaroxaban

	**SOC Cohort**		**Rivaroxaban Cohort**		
	**N=(192)**		**N=(64)**		
	**N/Mean**	**%/SD**	**N/Mean**	**%/SD**	**p-value**
**PE-related Clinical Outcomes during the 90-day follow-up period**					
Recurrent VTE	9	4.7%	2	3.1%	0.5935
Time to first VTE, days	47.6	24.6	29.5	26.2	0.3763
Major Bleeding	2	1.0%	0	0.0%	0.4124
Time to first Major Bleeding, days	5.00	2.8			
Death	3	1.6%	0	0.0%	0.3145
Time to Death, days	35.33	30.4			
**90-day Follow-up Diagnostic tests**					
CTA	63	32.8%	17	26.6%	0.3502
ECHO	14	7.3%	2	3.1%	0.233
VQ Scan	6	3.1%	1	1.6%	0.5068
Venous Doppler Ultrasound	56	29.2%	16	25.0%	0.5208

CTA: computed tomography angiography; DVT: deep vein thrombosis; ECHO: echocardiogram; PE: pulmonary embolism; SD: standard deviation; SOC: standard of care; VQ: lung ventilation/perfusion; VTE: venous thromboembolism

#### HRU and Costs

**Figure 2.**
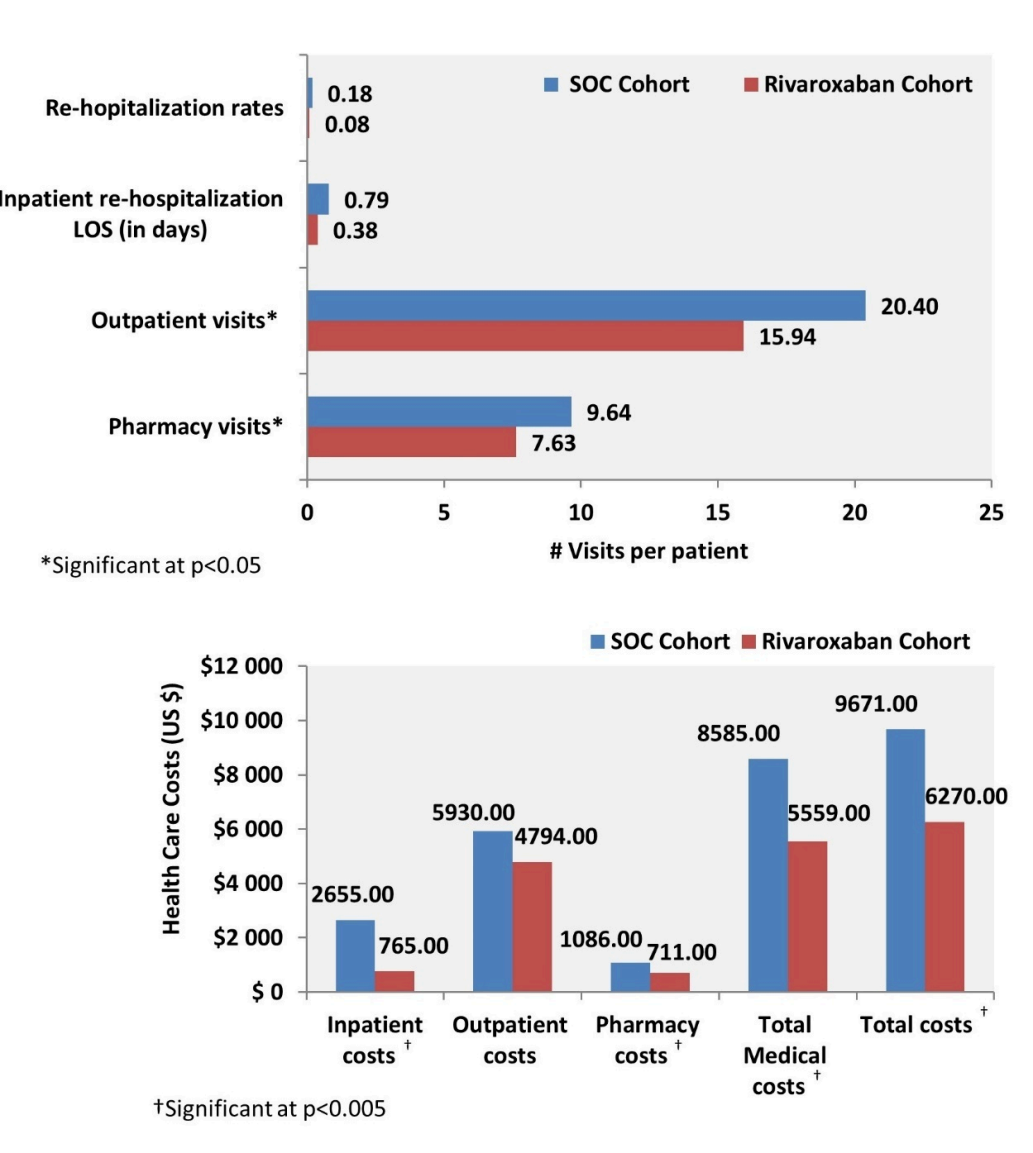
PSM-adjusted HRU and Costs among LRPE Patients Prescribed SOC Therapy versus Rivaroxaban During the 90-day Follow-up Period HRU: healthcare resource utilization; LOS: length of stay; LRPE: low-risk pulmonary embolism; PSM: propensity score matching; SOC: standard of care.

Compared to the SOC cohort, the rivaroxaban cohort had fewer outpatient (15.9 vs 20.4; p=0.0002) visits per patient and similar re-hospitalization rates (0.1 vs 0.2, p=0.1682; Figure 2). Also, rivaroxaban patients incurred lower inpatient ($765 vs $2655, p<0.0001), pharmacy ($711 vs $1086; p=0.0033), total medical ($5559 vs $8585; p=0.0026), and total costs ($6270 vs $9671; p=0.0027; Figure 2).

## Discussion

To our knowledge, this is the first study to evaluate the efficacy and cost implications of rivaroxaban use compared to SOC among LRPE patients in a real-world setting. We found that patients with LRPE prescribed rivaroxaban had lower HRU and ~1.5 times lower total healthcare costs than those prescribed SOC therapy. Our findings are consistent with several previous reports suggesting rivaroxaban could reduce healthcare costs by decreasing medication expenditures for injections and frequent INR measurement, which facilitates ambulatory treatment.^9,11,14,15,29^ The results of our study showed that the LRPE patients prescribed rivaroxaban had lower pharmacy costs. Other analyses of rivaroxaban versus heparin/warfarin in LRPE patients identified using sPESI score reported lower annual costs ($7234 vs $12 143)^30^ and a Premier database study that used the In-hospital Mortality for PE using Claims data (IMPACT) criteria to identify the LRPE patients, found patients prescribed rivaroxaban had ~1.5 times lower annual healthcare costs than SOC therapy.^31^ Ultimately, our results suggest that rivaroxaban can significantly reduce the economic burden of LRPE treatment.

We also note that 28.4% of PE patients in a VHA population can be stratified as low-risk. This finding may have implications in regards to potential hospital admission avoidance and is in agreement with analyses by Dentali et al in which 26.1% of PE patients were classified as low-risk per the sPESI criteria.^32^ Lefebvre et al has also suggested that the number of hospital admissions could be minimized as outpatient treatment is available for rivaroxaban due to oral administration.^10^ Since hospital costs are the greatest driver of total LRPE healthcare expenditures, validation of this finding could have an important financial impact in the overall management of PE.

The results of our study showed no differences in the index hospital LOS as well as the inpatient visits during the 90-day post-discharge date between the patients prescribed rivaroxaban or SOC therapy, with an average index LOS of 7 days in both the cohorts. This may have been the result of the small sample size limiting our ability to detect a difference in the hospital LOS, as several previous studies have shown that PE patients prescribed rivaroxaban had a shorter LOS, compared to warfarin patients, with a reduction ranging from 0.9-2 days.^9,11,14,15^ A possible explanation for the longer LOS in these other investigations is that clinicians need to observe an appropriate rise in the international normalized ratio (INR) prior to discharge in the warfarin cohort, which prolongs the LOS as compared to rivaroxaban.^11,15^ In a study conducted by Weeda et al in which the sample was restricted to only LRPE patients, rivaroxaban use was associated with even further index LOS reductions (~4 days).^30^ Despite no statistical difference in the index LOS, patients in the rivaroxaban cohort in our study were less likely to have HACs during index hospitalization. Despite the differences in study design, setting, and population, several previous studies have shown that patients with longer LOS have increased HACs.^33–35^ Additionally, the inpatient costs during the follow-up were lower in the rivaroxaban cohort despite the similar rate of inpatient visits. These results highlight the need for further research in a larger cohort of LRPE patients to understand the effect of rivaroxaban on inpatient LOS.

Our study evaluated outcomes in a 90-day follow-up period because this period is clinically significant with a higher prevalence of adverse outcomes including major bleeding, recurrent VTE, and death.^36^ The rationale was also justified by several previous studies on PE.^37,38^ However, the results of our study showed no differences between the cohorts regarding PE-related clinical outcomes such as recurrent VTE, major bleeding, and death during the 90-day follow-up period. This is consistent with other trials which reported lower or similar rates of major bleeding in the rivaroxaban as compared to warfarin cohorts.^22,39^ Although reported by others, we did not identify an increased risk of gastrointestinal bleeding with rivaroxaban.^40,41^ The results of our study are in agreement with previous clinical trials which conclude that rivaroxaban and SOC therapy had similar VTE recurrence, efficacy, and safety outcomes.^42^ Therefore, our analysis provides further support that rivaroxaban is an equally effective treatment option to SOC therapy for PE.

## Limitations

The findings from our study should be viewed in the context of its limitations. First, the study relied on retrospective claims data. While claims data are valuable for efficient and effective examination of healthcare outcomes, treatment patterns, and costs, they are collected for payment and not research. The presence of a diagnosis code on a medical claim is not a guarantee of the presence of disease as it may be incorrectly coded or included as a rule-out criterion rather than the actual disease. To make sure we did not include any rule-out PE diagnoses, PE patients were required to have an anticoagulant pharmacy claim during their hospital stay. Second, the presence of a claim for a filled prescription does not confirm that the medication was taken as prescribed, or at all. Also, prescriptions filled over-the-counter or provided as samples by the physician are not observed in claims data. Thus, the true number of medications prescribed may not be accurately recorded. Third, certain clinical and disease-specific parameters are not readily available in claims data that could affect study outcomes. It should also be noted that PSM adjustment cannot resolve problems due to imbalances in unmeasured factors. It is possible that there were unobserved variables that the PSM did not correct for in risk-adjusted tables. With the 1:3 matching, the standardized difference was higher (>10%) in some variables so a sensitivity analysis was performed with 1:1 PSM matching. However, similar results were reported so the 1:3 matching results are presented to achieve higher power. Additionally, due to a very small sample size in the rivaroxaban cohort, exact matching was not performed, and the results should be interpreted with caution. Future research with larger sample size should be conducted to validate our results. Finally, our study represented US data from a specific subpopulation (US veterans) who were mostly elderly men. Therefore, the general applicability of our findings to a civilian community population requires further study.

## Conclusions

LRPE patients prescribed rivaroxaban had similar LOS, and PE-related outcomes, but fewer HACs and incurred $3401 in lower total costs than patients who were prescribed the SOC. Therefore, our study provides evidence that an oral, single-drug approach with rivaroxaban may be a cost-effective alternative treatment option to SOC for LRPE patients.

## Source of Support:

This study was funded by Janssen Scientific Affairs, LLC.

## Author Contributions:

WFP, CIC, PW, GJF, CC, and JS conceptualized and designed the study. LW and OB verified and analyzed the data. All authors substantially contributed to the interpretation of the data and wrote the manuscript and/or substantially contributed to critical revisions of the intellectual content. All authors agreed to the final version of this manuscript.

## Conflict of Interest Disclosure:

WFP has received grants from Abbott, Alere, Banyan, Cardiorentis, Janssen, Portola, Pfizer, Roche, and ZS Pharma; is a consultant to Alere, Beckman, Boehringer-Ingelheim, Cardiorentis, Instrument Labs, Janssen, Phillips, Portola, Prevencio, Singulex, The Medicine's Company, and ZS Pharma; and also has ownership interests at the Comprehensive Research Associate LLC, Emergencies in Medicine LLC.

CIC has received grant funding and consulting fees from Janssen Scientific Affairs, LLC, Raritan, NJ and Bayer Pharma AG, Berlin, Germany.

PW receives speaker fees from Bayer Healthcare and Daiichi Sankyo, writing committee fees from Itreas, and grant support fees from Pfizer/BMS. [ORCID number 000-0002-8657-8326]

GJF has received research support from Novartis, Siemens, Pfizer, Portola, and PCORI; has advised Janssen Scientific Affairs, LLC; and receives speaker fees from Janssen.

CC and JS are employees of Janssen Scientific Affairs.

LW is an employee of STATinMED Research, which is a paid consultant to Janssen Scientific Affairs.

OB has no conflicts to declare.

## Acknowledgements:

The authors thank Sulena Shrestha and Sujana Borra of STATinMED Research for medical writing assistance.
